# Effectiveness of illness management and recovery (IMR) in the Netherlands: a randomised clinical trial

**DOI:** 10.1186/s12888-016-0774-0

**Published:** 2016-03-19

**Authors:** Bert-Jan Roosenschoon, Cornelis L. Mulder, Mathijs L. Deen, Jaap van Weeghel

**Affiliations:** Department of Psychiatry, Epidemiological and Social Psychiatric Research Institute, Erasmus University Medical Centre, ‘s-Gravendijkwal 230, Rotterdam, CE 3015 The Netherlands; Parnassia Psychiatric Institute The Hague, Kiwistraat 32, Den Haag, 2552 DH The Netherlands; Parnassia Psychiatric Institute, Bavo-Europoort, Prins Constantijnweg 48-54, Rotterdam, 3066 TA The Netherlands; Faculty of Social and Behavioral Sciences, Institute of Psychology, Leiden University, Wassenaarseweg 52, 2333 AK Leiden, The Netherlands; Tilburg School of Social and Behavioral Sciences, Department of TRANZO, Tilburg University, Warandelaan 2, Tilburg, 5037 AB The Netherlands; Parnassia Psychiatric Institute, Dijk en Duin, Oude Parklaan 125, Castricum, 1901 ZZ The Netherlands

**Keywords:** Illness management and recovery, IMR, Self management, Severe mental illness, Schizophrenia, Training, Recovery, Personal goals

## Abstract

**Background:**

Illness Management and Recovery (IMR) is intended to provide a structured psychosocial programme that helps to manage the disabling effects of severe mental illnesses such as schizophrenia and bipolar disorders. It is curriculum based and aims to improve different aspects of illness management and recovery through interventions such as goal-setting, psycho-education, coping and social skills training. Its overall aim is to improve illness outcomes and support subjective and objective recovery. To date there have been four RCTs on IMR; as these yielded mixed results, further research is needed. Our hypotheses aim to test the interrelatedness assumed in Mueser’s Conceptual Framework for IMR for the many aspects of illness management, illness management outcomes and recovery.

**Methods/design:**

This randomised multi-centre, single-blinded clinical trial is intended to compare IMR with treatment as usual for 200 outpatient clients with a severe and persistent mental illness (SMI). We will investigate whether IMR leads to better illness management, fewer symptoms and fewer relapses, and also to better subjective and objective recovery. The primary outcome measure is the score on the client version of the Illness Management and Recovery Scale. Secondary outcome measures are the clinician version of the Illness Management and Recovery scale, measures of illness management, coping, symptoms, the number of relapses, and measures of recovery. Measurement will take place before randomisation, and 12 and 18 months after randomisation.

**Discussion:**

Overall, our study has the following strengths: 1.) our use of an RCT design in a country where the earlier RCTs on IMR were not conducted; 2.) the fact that participants will consist not only of people with a diagnosis of schizophrenia, but also of those with various types of SMI; 3.) our inclusion of 200 participants; and 4.) the fact that we will explore the working mechanisms described in Mueser’s Conceptual Framework for IMR. Finally, 5.) because the RCT will be conducted in everyday clinical practice, we believe that the generalisability of our results will be good.

**Trial registration:**

The Netherlands National Trial Register (identifier: NTR 5033). Date registered: 13 January 2015.

## Background

### Introduction

Due to the disabling effects of their illness, it is hard for people with severe and persistent mental illnesses (SMI) such as schizophrenia or a bipolar disorder to participate fully in society. Though they have the same aspirations as other people, these wishes are harder to realize, due not only to their illness, but also to barriers within society (such as stigma) [1]. Mental-health care should therefore include interventions that support individual recovery and contribute to self-determination and well-being, and also to skills for illness self-management and for fulfilling valued roles in domains such as work, social connections and housing [2, 3].

In recent years, various psychosocial interventions have been developed to support recovery, such as the Cognitive Adaptation Training (CAT) [4], the Wellness Recovery Action Plan (WRAP) [5] and the Boston Psychiatric Rehabilitation (PR) Approach [6].

Over the last decade, a promising new programme for people with SMI has been developed: Illness Management and Recovery (IMR) [2], a programme that combines psychosocial interventions such as psycho-education with aspects of cognitive behavioural therapy, skills training, peer support and rehabilitation. These interventions aim to help participants gain greater control of their problems through illness management, and also to support their recovery.

The IMR programme was based on an empirical review of the research literature on teaching illness self-management strategies to people with SMI [7]. It was also part of the National Implementing Evidence-Based Practices Project in the U.S. [8, 9]. In themselves, the different parts of the IMR programme were not new; the newness lay in offering them as an integrated package.

The theoretical foundation of IMR rests on two models. The first, the trans-theoretical model, holds that people are more motivated to acquire new behaviour if the types of intervention are adjusted to the stage of change they are in. This makes it is easier for people to become aware of their problems, to take decisions, and to implement and sustain change [10, 11].

The second model is the stress-vulnerability model [2], which holds not only that mental health problems originate from the interaction between biological vulnerability and sources of stress in the environment, but also that people differ in their coping ability [12, 13]. In line with the stress-vulnerability model, IMR -trainers need to teach participants the basics of illness (self-) management—enabling them, for example, to reduce substance use, improve adherence to medication, increase coping and social support, and become involved in meaningful activities. This may improve illness outcomes such as symptoms, relapse, and hospitalisation.

**Fig. 1:**
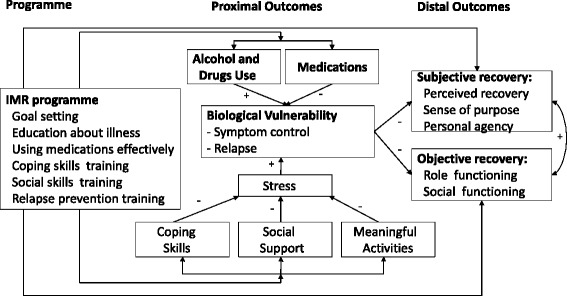
Conceptual Framework for the Illness Management and Recovery programme of Mueser et al. [2]

By combining better illness management with the pursuit of personal goals, progress may be made towards recovery. In Mueser’s model, the IMR programme may also lead directly to recovery, though it should be noted that Mueser differentiates between subjective recovery (perceived recovery, sense of purpose, and personal agency) and objective recovery (role and social functioning) [2] (see Fig. 1).

IMR is seen as an Evidence Based Practice (EBP) by the researchers who designed it and by the Substance Abuse and Mental Health Services Administration (SAMSHA) in the U.S., who reasoned that its components were evidence based [7, 9]. However, research on the overall programme was needed.

In a review conducted in 2011, it appeared that three randomised controlled trials (RCTs), three quasi-controlled trials and three pre-post trials on the overall programme had been conducted [14]. The results of one further RCT have also been published [15]. The RCTs differed from each other with regard to setting, participant number and diagnoses, the length and format of IMR, the trainers’ training and their qualifications, the intensity of supervision of the trainers, the number and timing of measurements, the type of control group and the fidelity of the implementation of IMR. The results of the four RCTs are presented in Table 1.

**Table 1:** Results of four completed RCTs on IMR (by study^a,b^)

	Hasson-Ohayon et al. 2007 [17]	Levitt et al. 2009 [18]	Färdig et al. 2011 [16]	Salyers et al. 2014 [15]
Consumer report				
IMR Scale.	NS	.36	.29	NS
Patient activation				NS
Recovery			NS	NS
Hope				NS
Coping	NS		.14–.19^c^	
Knowledge about mental illness	.14^d^			
Psychiatric symptoms		NS		
Quality of life, community functioning, and social support	NS		NS	
Medication adherence				NS
Clinician report				
IMR Scale	.28	.39	.34	
Quality of life, community functioning, and social support		.52		
Substance abuse		NS		
Observer-rated psychiatric symptoms		−.20	.38	NS
Interviewer−rated Quality of Life				NS
Objective outcome				
Hospitalizations and emergency	NS	NS	NS	NS
Visits				
Employment		NS		
Medication dosage			NS	
Inpatient admission				NS
Inpatient psychiatric admission				NS
Length of inpatient stay				NS
Length of inpatient psychiatric stay				NS

^a^The results of the studies of Hasson-Ohayon et al. [17], Levitt et al. [18], Färdig et al. [16] and notes b, c and d are taken from McGuire et al. [14]

^b^Results reflect comparisons from baseline to the longest follow-up period. Studies reported only one scale for each category. Only significant (p,.05) effect sizes (Cohen’s d) are reported. Effect sizes for Färdig et al. [16] are reported as η^2^. A blank cell indicates that the variable was not measured. NS: not significant

^c^ Range from the four of eight subscales of the Ways of Coping Scale with significant results

^d^ Knowledge and goals subscale of the consumer-reported IMR Scale

The first three RCTs compared IMR with care as usual (CAU) [16–18]. On the overall score of the client version of the Illness Management and Recovery Scale (IMRS [19], two of these studies showed significantly positive results for clients assigned to IMR, with respective effect sizes of .36 [18] and .29 [16]. The other study found significant improvement in IMRS scores only if the analyses were limited to sites with high IMR fidelity [17]. On the overall score of the clinician version of the IMRS, all three of these studies showed significantly positive results for clients assigned to IMR, with respective effect sizes of .28 [17], .39 [18] and .34 [14, 16]. On the overall score of both the client version and the clinician version of the IMRS, the more recent RCT of Salyers—in which IMR was tested against an active control group [15]—showed no significant differences between the experimental and control group.

In all four studies, additional significantly positive results for IMR were found on client-reported knowledge in one study [17], on client-reported coping in another study [16], on clinician-reported quality of life in a third study [18], and on observer-rated psychiatric symptoms in two of these studies [16, 18]. These results were either not found in the other RCTs, or the domains in question were not measured. No significant outcomes were found on objective outcomes such as medication dosage, employment, or hospitalisations and emergency visits. While Salyers found no significant differences between IMR and the active control group on any of the domains measured (Table 1), the respective participation rates in the two interventions were only 28 and 17 % [15].

These mixed results indicate the need for more research.

We aim to use an RCT design to study the effects of IMR in a Dutch context. On both of the IMR scales, we expect positive results of the sort found in the earlier studies that used CAU as a control [16–18]. We also hope to gain additional information with regard to symptoms, coping and recovery, on which the earlier results differed. By using different outcome measures of illness management, illness outcomes, and recovery, our study will provide a thorough measurement of the effects of IMR.

Before designing the main study, we conducted a pilot study to explore the feasibility of an RCT and to provide practical guidelines for its implementation. This study suggested that an RCT on IMR was feasible: not only could sufficient participants be recruited for all six IMR-groups, which could be established with good mean fidelity, but support for a broader implementation of IMR could also be identified [20].

### Research aims

The aim of this study is to compare the effectiveness of the IMR programme with that of CAU in people with SMI. Specifically, we wish to compare the effects of ‘IMR + CAU’ with those of ‘CAU only’ on illness management and recovery.

### Hypotheses

We have two primary hypotheses and four secondary hypotheses. The first primary hypothesis is that IMR + CAU (IMR offered in group format) leads to better illness management and to fewer symptoms and relapses than CAU only. The second primary hypothesis is that IMR + CAU leads to better ‘subjective’ and ‘objective’ recovery than CAU only. We thus expect a condition x time interaction effect of the IMR + CAU group over the CAU-only group.

The first secondary hypothesis is that the cost-utility of IMR + CAU is better than that of CAU.

We intend to explore the working mechanisms of IMR by testing the second secondary hypothesis that better illness management (i.e. getting greater psychiatric insight, better coping, more social support, less addiction and better service engagement) leads to fewer symptoms and relapses. We also intend to test the third secondary hypothesis that better ‘distal outcomes’ (i.e. recovery, see Fig. 1) result from a combination of better ‘proximal outcomes’ (i.e. better illness management and fewer symptoms and relapses) and progress on personal goals [2]. Finally, we expect that any improvement resulting from IMR + CAU will be associated with the fidelity of IMR implementation (fourth secondary hypothesis).

## Design

This research project entails a randomised controlled trial in which clients who provide written informed consent will be assigned to the experimental condition (IMR) or the control group. The experimental group will consist of clients who participate in the IMR programme (which will be offered in a group format) and also get CAU. The control group will receive CAU.

Measurement is planned to take place before randomisation and at 12 and 18 months after randomisation.

### Participants

The participants in the study will be adult SMI outpatient clients aged between 18 and 65 who have given written informed consent. There will be three exclusion criteria: having already participated in IMR training, being unable to give informed consent due to mental incompetence, and insufficient knowledge of the Dutch language.

Most participants will have a psychotic disorder or a schizoaffective disorder with or without comorbid disorders such as substance abuse and personality disorders.

#### Recruitment procedure

Participants will be recruited from 14 community mental health teams at 11 branches of two mental health institutions in the greater Rotterdam area (the Netherlands). Eight teams throughout the city of Rotterdam serve an urban area of approximately 1.2 million inhabitants, one team serves a more rural environment to the west of Rotterdam, and five teams serve the neighbouring city of Dordrecht (120,000 inhabitants) and four smaller communities of 20,000–35,000 inhabitants.

During interviews with an assistant researcher, the caseloads of all clinicians in these teams will be assessed for their potential suitability for IMR. The clinicians will ask selected clients to participate in IMR and about their willingness to be informed about the study. If clients express interest and agree to be contacted by an assistant researcher for more detailed information, the assistant researcher will explain the research goals, randomisation procedure and the three times of measurement. Clients will then be asked whether they want to participate in the study. After written informed consent has been given, the baseline interview will be followed by randomisation.

#### Intervention

In essence, IMR is a structured training consisting of 11 modules, practitioner guides and handouts for participants. The 11 modules are 1.) Recovery Strategies, 2.) Practical Facts about Mental Illness, 3.) Stress-Vulnerability Model, 4.) Building Social Support, 5.) Using Medication Effectively, 6.) Drug and Alcohol Use and Treatment Strategies, 7.) Reducing Relapses, 8.) Coping with Stress, 9.) Coping with Problems and Persistent Symptoms, 10.) Getting Your Needs Met in the Mental-health System, and 11.) Health for You.

The IMR training will be given at the participating institutes in a group format with weekly sessions. The original American text [19] has been translated into Dutch and will be adapted to the Dutch context if necessary.

During the first module, participants will decide which personal goals they want to work on during the programme. For the first half of each 90-min session, some will work on their goals in the group. During the second half, all participants will work with the help of the handouts on the subjects of the modules.

Our pilot study showed that most modules will require an average of three to four sessions. Each IMR group will be guided by two trainers, who will use 1.) motivation-enhancement strategies such as conveying confidence and exploring the pros & cons of change; 2.) educational strategies (psycho-education) such as interactive teaching, breaking down information, and checking for understanding; and 3.) cognitive-behavioural techniques such as shaping, modelling and role playing. Peer-group support and coping & social skills training are integral to IMR. Homework assignments will be provided. Workbooks and homework assignments can be accessed through an e-health module.

### IMR model fidelity

To promote the fidelity of the intervention, the trainers will be experienced clinicians (mostly psychiatric nurses) who will receive a 2-day course in teaching IMR before the study starts and who will attend supervision once every 2 weeks for 2 h. Twice a year, all IMR trainers will come together for a morning or afternoon of additional training.

To measure whether the IMR-programme is implemented according to the original intent, the researchers will determine model fidelity using the IMR fidelity scale and the IMR General Organisational Index (GOI) [19, 21]. These scales have been translated, and the principal investigator was trained in their use by two American specialists (M.P. Salyers, Ph.D. and A. Mc Guire, Ph.D, both of UIPUI, University of Indianapolis USA). We will also use the Illness Management and Recovery Treatment Integrity Scale (IT-IS) [22], which gives more precise information on the quality of the trainers’ interventions. These fidelity scales will be applied by the principal researcher and a research assistant, scoring independently according to a protocol. For one team, fidelity measurement takes almost a day, and consists of interviews with two participants and the two trainers, in addition to observation of one session, and checking forms. The researchers will give periodic feedback in the supervision groups on the results of the fidelity measurements.

### Care as usual

CAU will involve outpatient case management, medication and rehabilitation services, with no restrictions on anything. The usual frequency of treatment contacts is 1.) one face-to-face contact with a mental health nurse every 2 weeks, and 2.) contact with a psychiatrist when indicated and no less than once a year. To indicate what CAU actually comprised, we will register the use of care in the control group.

### Instruments

Data will be collected on bio-demographic variables, illness management, illness outcomes and recovery. For an overview of instruments, data sources and times of measurement, see Table 2.

**Table 2:** Instruments, data sources and times of measurement

Domain	Aspect	Instrument	Data source			Time of measurement		
			Patient file	Patient	Clinician	M1	M2	M3
Bio-demographic data			x	x	x	x		
Diagnosis			x		x	x		
Illness management	Coping	CSES		x		x	x	x
	Social Support	MSPSS		x		x	x	x
	Treatment compliance	SES			x	x	x	x
	Insight	Insight Scale (IS)		x		x	x	x
	Addiction	Item 24 of the ASI		x		x	x	x
Illness management + illness outcomes		IMR scale, patient version		x		x	x	x
		IMR scale, clinician version			x	x	x	x
illness outcomes	Symptoms	BSI		x		x	x	x
	Hospitalisations	Records of Mental Health Institution				x	x	x
	Health complaints and functional limitations	EQ-5D		x		x	x	x
Subjective recovery	Generic	MHRM		x		x	x	x
	Self-Stigma	ISMI		x		x	x	x
	Self-Esteem	SERS-SF		x		x	x	x
	Goals	Granholm’s Goals Template			x	x	x	x
	Satisfaction	One question of the ROM		x		x	x	x
Objective recovery	Social Functioning	The SF Scale		x		x	x	x
Fidelity		IMR-fidelity scale, GOI;	x	x	x	Between M1 & M2		
		IT-IS scale						

#### Bio-demographic variables

Bio-demographic variables and psychiatric history will be collected at baseline during the interviews with the clients and the clinician; they will be checked in the electronic client files. The diagnoses were made earlier on the basis of a clinical interview by psychiatrists on the community mental health team, and will be collected during the interviews with clinicians and checked in the electronic client files.

#### Illness management and illness outcomes

The primary outcome measure is the self-rated IMRS [23, 24] with 15 items completed by clients themselves. One of the secondary outcome measures is the clinician-rated IMRS [23, 24]. The IMR scales have good validity and moderate reliability [25, 26]. To identify and correct discrepancies, the Dutch translation has been independently back-translated into English and compared with the original version. Evidence has been provided for the reliability and validity of this Dutch version [27].

These two IMR scales are not unidimensional measures [25, 28]. Three factors were found: ‘Coping with Illness Outcome’, ‘Knowledge and Goals’, and ‘Effective Medication Use/Reduced Alcohol and Drug Abuse’ [25]. In terms of Mueser’s framework, the items of these two IMR scales mainly concern aspects of illness management and illness outcomes.

##### Additional illness-management scales

Given the limited number of items in the IMR scales, we will also assess illness management using other validated and more comprehensive scales, assessing coping, social support, treatment compliance, insight into illness, and problems with alcohol and drugs. To measure these secondary outcome variables, we will use the following:

The coping self-efficacy scale (CSES [29]), 13-items. This scale has good internal reliability and three factors: using problem-focused coping (six items), stopping unpleasant emotions and thoughts (four items), and getting support from friends and family (three items). Internal consistency and test–retest reliability are strong for all three factors.

The Multidimensional Scale of Perceived Social Support (MSPSS [30]),12 items. The MSPSS was found to have good internal reliability and a strong factorial validity according to a three-subscale structure: Family, Friends, and Significant Others.

The Service Engagement Scale (SES [31]), 14 Items. The scale has high internal consistency and retest reliability and four sub-scales: availability, collaboration, help-seeking, and treatment adherence.

The Insight Scale (IS [32]), 8 items. This scale captures three dimensions of insight: perceived need for treatment, awareness of illness, and re-labelling of symptoms as pathological. The psychometric properties of the scale have been called excellent [33].

One item (item 24) of the Addiction Severity Index (ASI [34, 35]) will be used to measure the extent to which respondents have been bothered in the past 30 days by problems with a.) alcohol, or b.) drugs.

##### Illness outcomes: symptoms and relapses

The secondary outcomes on illness-management outcomes are symptoms, health complaints & functional limitations, and relapses. These topics will be measured with the following:

The Brief Symptom Inventory (BSI [36–38]), 53 items. The authors report good validity, internal consistency and test-retest reliability for the nine dimensions: Psychoticism, Depression, Somatization, Phobic Anxiety, Obsessive Compulsive, Interpersonal Sensitivity, Anxiety, Hostility, and Paranoid Ideation; and also good test-retest reliability for the three Global Indices: global severity index (GSI), positive symptom total (PST), and positive symptom distress index (PSDI).

The EQ-5D [39], five items. This scale measures primarily health complaints (in a broad sense) and functional limitations. The EQ-5D is the Euro-QOL self-report scale and has good psychometric properties.

The number of relapses will be operationalised as the number and duration of hospital admissions and number of emergency-department visits.

#### Recovery

In Mueser’s conceptual framework [2], the concept of recovery is differentiated into subjective recovery and objective recovery. The concept of subjective recovery is complex. Mueser divides subjective recovery into perceived recovery, sense of purpose and personal agency, and divides objective recovery into social and role functioning. As secondary outcome measures we will use five scales to assess subjective recovery and one longer scale for objective recovery.

##### Subjective recovery

We will assess subjective recovery using a specific recovery scale, and by assessing internal stigma, quality of life, self-esteem and life-goals on the basis of the following:

The Mental Health Recovery Measure (MHRM [40, 41]; authorized Dutch translation [42]), 30 items. The Dutch version is a reliable measure in terms of internal consistency; convergent and divergent validity are generally acceptable [43]. The authors differentiate three subscales; ‘self-empowerment’ (13 items), ‘learning and new potentials’ (15 items) and ‘spirituality’ (2 items).

The Internal Stigma of Mental Illness (ISMI [44]); 29 items. This scale is designed to measure the subjective experience of stigma, with subscales measuring Alienation, Stereotype Endorsement, Perceived Discrimination, Social Withdrawal and Stigma Resistance. The ISMI has high internal consistency and test-retest reliability. Construct validity and divergent validity were supported by comparisons with scales measuring related constructs.

One item of the Quality of Life section of the Cumulative Needs for Care Monitor (CNCM [45]), which asks: ‘’Can you tell me how satisfied you are with your life as a whole?” This item correlates strongly with the total score of this Quality of Life section.

The Self-Esteem Rating Scale-Short Form (SERS-SF [46]), 20 items. This scale has two subscales: positive and negative self-esteem. It also has good internal consistency, good test-retest reliability and adequate convergent validity for people with schizophrenia.

In line with Muesers’ conceptual framework, we consider improving on personal goals to be a mediator variable between illness self-management and recovery ([2], p. S35). We will measure this with Granholm’s Goals Template [47], an instrument for Goal Attainment Scaling that measures progress towards goals in ten life domains: employment, housing, relationships, school, self-care; leisure activities, addictions, money-management, independence using transportation, and general template. We have developed a very short manual for this scale, whose psychometric properties have not yet been investigated.

##### Objective recovery

Objective recovery will be assessed using the Social Functioning Scale [48]. Mueser defines social and role functioning as aspects of objective recovery. Both are covered with this scale, which has 19 items and the following seven dimensions: social withdrawal, relationships, social activities, recreational activities, independence (competence), independence (performance), and employment. This scale has been described as reliable, valid, sensitive and responsive to change.

#### Cost-utility

The number and duration of outpatient treatment contacts and inpatient days will be calculated in cost in Euro’s. They are related with changes in quality of life measured with the EQ-5D [39]. Cost-utility can be calculated by transforming scores on the EQ-5D into so-called QALYs [49]. To calculate cost-utility, only the cost of health care consumption is included, not social costs such as rent, benefits, etc.

### Blinding

Thirteen of the above scales are self-report scales, and two (IMRS-clinician version and SES) will be rated by non-blinded clinicians who will not be involved in the IMR training. Granholm’s Goals Template will be rated by the blinded research assistants in an interview with the clinician.

At the second and third measurements, the measurement procedure will be single-blinded: the research assistants will not know whether a client is participating in the experimental condition or the control condition; this procedure will preclude experimenter’s bias. To ensure this, clients and clinicians alike will be instructed before the interview not to inform the interviewer in any way about the client’s condition. At the end of each interview, interviewers will answer the question whether they have found out which condition the clients are in.

### Sample size

On the basis of the effect sizes of the studies by Hasson-Ohayon et al. (2007), Levitt et al. (2009) and Färdig et al. (2011) [16–18], we anticipate a medium effect size of 0.40 on the primary outcome variable (self-rated IMR scale, Mueser et al. 2004, 2005 [23, 24]). On the basis of the power analyses with three times of measurement (mixed models), equal allocation to the experimental and control groups, a power of .80, alpha at 0.05, and an effect size of .40, it will be necessary to randomize 148 clients: 74 to the experimental condition and 74 to the CAU group [50, 51].

### Randomisation

Because IMR is a relatively long-term care programme—in our pilot study at least 8 months at one session per week—the drop-out from treatment may be relatively high. Other studies had 29 % [17], 15 % [18]; 51 % [20], and 72 % [15]. Due to the 50 % drop-out rate expected from treatment in the experimental condition (a pessimistic estimate), we have chosen to allocate more clients to the experimental condition (IMR) than to the CAU group (proportions of 3:2). We will randomise 200 clients (on the basis of a conservative calculation): 120 to the experimental condition and 80 to the CAU group.

Randomisation will be stratified by team. To ensure the right distribution of clients per team in the two groups, envelopes containing five lots per team will be used each time. For this we will use a randomisation plan from http://www.randomization.com. After completing the baseline interviews, the principal investigator will allocate participants to the experimental group or control group.

### Statistical analysis

The analyses will be based on the intention-to-treat principle. Generalized linear mixed models will be used to investigate group difference between the experimental and control conditions [52, 53], with times of measurement nested within study participants. As well as the main effects of time and condition, we will investigate whether there is a time x condition interaction effect with respect to symptoms, health complaints, number of relapses and recovery. Because number of relapses is a count variable, the analysis will use a mixed model for the poisson or negative binomial distribution. We will also test whether the addition of a random effect for the time variable results in a better model fit in each of the analyses. For the recovery outcome, we will investigate the influence of illness management, symptoms, relapses and progress on personal goals, as well as their interaction with time. Akaike’s Information Criterion (AIC [54]) will be used for statistical model selection in these analyses.

To explore associations between illness-management outcomes and recovery outcomes, we will use structural equation modelling. To investigate the associations between improvement of symptoms, recovery and fidelity (within IMR + CAU), linear mixed models will be used, with clients nested within IMR groups. As well as a fixed effect for the trainer’s fidelity score, a random intercept at the group level will be incorporated. Before testing the first and second primary hypotheses, we will test the fourth secondary hypothesis: ‘we expect that any improvement resulting from IMR + CAU will be associated with the fidelity with which IMR is implemented.’ If fidelity has an effect, it will be included as a covariate in the subsequent analyses.

Clients will be classified as completers of the study if they have finished all three interviews. After the intention-to-treat analyses, we will perform secondary analyses to measure the effects in clients who have completed IMR.

#### Ethical considerations

The study protocol, information brochure and informed consent form were consistent with the declaration of Helsinki and approved by the Dutch Union of Medical-Ethic Trial Committees for mental health organisations (registration number of the Dutch National Trial Register NTR 5033 http://www.trialregister.nl, CCMO-no. NL 38605.078.12).

## Discussion

As the four RCTs conducted on IMR have yielded mixed results, more research is needed. We therefore aim to compare the effectiveness of the IMR programme with that of CAU in people with SMI. Our first primary hypothesis is that IMR + CAU will lead to better illness management and fewer symptoms and relapses than CAU only. The second primary hypothesis is that IMR + CAU will lead to better ‘subjective’ and ‘objective’ recovery than CAU only. The first secondary hypothesis is that the cost-utility of IMR + CAU will be better than that of CAU. We intend to explore the working mechanisms of IMR by testing a second secondary hypothesis that better illness management will lead to fewer symptoms and relapses. We also intend to test a third secondary hypothesis that better ‘distal outcomes’ (i.e. recovery) will result from a combination of better ‘proximal outcomes’ (i.e. better illness management and fewer symptoms and relapses) and progress on personal goals. Finally, in our fourth secondary hypothesis, we expect that any improvement resulting from IMR + CAU will be associated with the fidelity with which IMR is implemented.

Our study has various strengths and limitations.

### Strengths

The first strength is that our use of different outcome measures for each domain will provide a thorough measurement of the effects of IMR on illness management and on subjective and objective recovery. And as well as exploring the contribution made by various illness-management variables to illness outcomes, we will also explore the contribution that progress on personal goals makes to subjective and objective recovery. In this way we will be able to analyse the working mechanisms of Mueser’s conceptual framework [2].

Other strengths of our study are that it will be conducted in another country than the earlier RCTs. By including 200 participants with various types of SMI, it will also have a relatively large sample size. The numbers of participants in the other completed RCTs were 210 [17], 104 [18], 41 [16] and 118 [15]. As the study will be conducted in the everyday clinical practice of the two participating mental health institutions, the generalisability of its results will be good. The measurement procedure at the second and third time of measurement will be single blinded.

### Limitations

Firstly, no structured diagnostic interview will be used to confirm the DSM-IV diagnosis. Practical considerations underlay our choice of the clinical diagnosis reported by clinicians on the basis of the medical records; due to the limited relevance of a DSM-IV diagnosis to the present study, this seems to be sufficient.

Another limitation is the chance of recruitment bias. As we consider clinicians to be responsible for treatment, it will be they who suggest that particular clients participate in IMR, and who inform these clients about the study. Although an interview with an assistant researcher will be used to screen all clinicians’ total caseloads for their potential suitability for IMR, and although the clinicians are indeed supposed to ask all selected clients to participate in IMR and to inform them about the study, some clinicians may prefer certain clients for participation in IMR—those whose functioning is better, for example. Some of these selected clients are therefore likely to participate in this study. We will explore which criteria the clinicians use to select participants for IMR.

A further limitation is that the clinicians who score two questionnaires will not be blind for the condition. On the other hand, they will not be involved in the IMR training. Neither, due to the use of self-score questionnaires, will they be blind for the clients.

The use of multiple scales to assess aspects of objective and subjective recovery is a limitation, as multiple testing may introduce positive findings. However, given the comprehensive nature of the IMR model and training programme, we felt that it would not be enough to use IMR scales alone [23, 24], and that a more comprehensive set of scales was needed.

By limiting the cost-utility analyses to health care consumption alone, we will not be able to include potential reductions in social costs, such as those that might occur if IMR participants manage their symptoms well enough to be able to work. But a full assessment of costs is beyond the scope of this study.

Finally, IMR will be offered in our study in a group format, and will be compared with care as usual, which will largely involve an individual format. Some of the possible effect in a group format may be attributable to peer-group support.

If evidence provides support for IMR, IMR may be recommended as part of the guidelines for SMI care, and be implemented more broadly. This will enable it to meet a need for a structured psychosocial intervention that supports illness management and recovery.

## Abbreviations

AIC: Akaike’s Information Criterion
ASI: Addiction Severity Index
BSI: The Brief Symptom Inventory
CAT: cognitive adaptation training
CAU: care as usual
CNCM: The Cumulative Needs for Care Monitor
CSES: the coping self-efficacy scale
EBP: Evidence Based Practice
EQ-5D: Euro-Qol 5D
IMR: illness management and recovery
IMRS: illness management and recovery scale
IS: The Insight Scale
ISMI: The Internal Stigma of Mental Illness
IT-IS: Illness Management and Recovery Treatment Integrity Scale
MHRM: The Mental Health Recovery Measure
MSPSS: The Multidimensional Scale of Perceived Social Support
PR: Boston psychiatric rehabilitation approach
QUALYs: Quality adjusted life years
RCT: randomised clinical trial
SAMSHA: Substance Abuse and Mental Health Services Administration
SERS-SF: The Self-Esteem Rating Scale-Short Form
SES: The Service Engagement Scale
SMI: serious mental illness
U.S.: United States of America
WRAP: wellness recovery action plan
